# Comparison of Antibodies Hydrolyzing Myelin Basic Protein from the Cerebrospinal Fluid and Serum of Patients with Multiple Sclerosis

**DOI:** 10.1371/journal.pone.0107807

**Published:** 2014-09-29

**Authors:** Visilii B. Doronin, Taisiya A. Parkhomenko, Massimiliano Castellazzi, Marina Padroni, Michela Pastore, Valentina N. Buneva, Enrico Granieri, Georgy A. Nevinsky

**Affiliations:** 1 Novosibirsk Medical University, Ministry of Public Health of Russian Federation, Novosibirsk, Russia; 2 Institute of Chemical Biology and Fundamental Medicine, Russian Academy of Sciences, Siberian Division, Novosibirsk, Russia; 3 Multiple Sclerosis Center, Department of Neurology, Ferrara University, Ferrara, Italy; 4 Novosibirsk State University, Novosibirsk, Russia; University of Oulu, Finland

## Abstract

It was found that antibodies (Abs) against myelin basic protein (MBP) are the major components of the antibody response in multiple sclerosis (MS) patients. We have recently shown that IgGs from sera of MS patients are active in the hydrolysis of MBP. However, in literature there are no available data concerning possible MBP-hydrolyzing Abs in cerebrospinal fluid (CSF) of MS patients. We have shown that the average content of IgGs in their sera is about 195-fold higher than that in their CSF. Here we have compared, for the first time, the average content of lambda- and kappa-IgGs as well as IgGs of four different subclasses (IgG1-IgG4) in CSF and sera of MS patients. The average relative content of lambda-IgGs and kappa –IgGs in the case of CSFs (8.0 and 92.0%) and sera (12.3 and 87.7%) are comparable, while IgG1, IgG2, IgG3, and IgG4: CSF - 40.4, 49.0, 8.2, and 2.5% of total IgGs, respectively and the sera - 53.6, 36.0, 5.6, and 4.8%, decreased in different order. Electrophoretically and immunologically homogeneous IgGs were obtained by sequential affinity chromatography of the CSF proteins on protein G-Sepharose and FPLC gel filtration. We present first evidence showing that IgGs from CSF efficiently hydrolyze MBP and that their average specific catalytic activity is unpredictably ∼54-fold higher than that of Abs from sera of the same MS patients. Some possible reasons of these findings are discussed. We suggest that anti-MBP abzymes of CSF may promote important neuropathologic mechanisms in this chronic inflammatory disorder and in MS pathogenesis development.

## Introduction

Artificial abzymes (catalytic Abs against transition state analogues of chemical reactions) and natural abzymes are novel biological catalysts that have attracted a lot of interest in recent years (reviewed in [1]–[5]). Artificial abzymes are abzymes against analogs of transition states of catalytic reactions [1]–[5] or antiidiotypic Abs induced by a primary antigen, which may show some of their features including the catalytic activity (for review also see [6]–[11]). During the past two decades it has become clear that auto-antibodies (auto-Abs) from sera of patients with different autoimmune diseases can possess enzymatic activities and that their occurrence is a distinctive feature of autoimmune diseases (reviewed in [11]–[14]). Different abzymes may play a significant role in forming specific pathogenic patterns and clinical settings in different autoimmune conditions through their broadened auto-Ab properties. Patients with autoimmune diseases produce Abs to nucleoprotein complexes, DNA and enzymes that participate in nucleic acid metabolism [11]–[14]. In autoimmune diseases, there can be a spontaneous induction of anti-idiotypic antibodies, which are Abs elicited by a primary antigen, including some with catalytic activity, or a transition from polyreactive catalytic activity to an autoantigen-directed activity. Natural abzymes hydrolyzing DNA, RNA, polysaccharides, oligopeptides, and proteins are present in the serum of patients with several autoimmune and viral diseases (reviewed in [11]–[14]). Healthy humans do not develop abzymes with detectable DNase and RNase activities, their levels being usually on the borderline of sensitivity of the detection methods [11]–[14].

Multiple sclerosis (MS) is a chronic demyelinating pathology of the central nervous system presenting a serious medical and social problem. Its etiology remains unclear, and the most valid theory of its pathogenesis assigns the main role in the destruction of the myelin-proteolipid shell of axons to inflammation related to autoimmune reactions ([15], and refs therein). Although the T-cell immune system plays a leading role in MS pathogenesis, the normal functioning of the B-cell system is also important for the development of the disease. An enhanced synthesis of immunoglobulins (usually IgGs), their free light chains and of a polyspecific DNA binding Abs interacting with phospholipids can be observed in MS patients [15].

It was shown, that myelin basic protein-component 1 (MBP-C1) from MS tissue undergoes autocatalytic cleavage at slightly alkaline pH [16]. Importantly, one of the major peptides released contained the immunodominant epitope. The cleavage reaction was not inhibited by protease inhibitors, except for phenylmethanesulfonyl fluoride, a serine protease inhibitor.

It has recently been shown that myelin basic protein (MBP)-hydrolyzing activity is an intrinsic property of IgGs, IgMs, and IgAs from sera of MS patients [14], [17]–[21]. In addition, it was shown that MS IgGs containing lambda (λ-IgGs) and kappa (κ-IgGs) light chains as well as IgGs of all four subclasses (IgG1-IgG4) efficiently hydrolyze MBP [20]. Recognition and degradation of MBP peptides by serum auto-Abs were confirmed as a novel biomarker for MS [22]. The established MS drug Copaxone appears to be a specific inhibitor of MBP-hydrolyzing abzyme activity [22]. Taking these observations into account, the analysis of relative concentrations of proteins and MBP-hydrolyzing abzymes in the cerebrospinal fluid (CSF) of MS patients is of special interest.

In the present study we have for the first time compared a relative content of total protein, λ-IgGs and κ-IgGs as well as IgGs of all four subclasses (IgG1-IgG4) in sera and CSFs of MS patients. Using different approaches, we provide, for the first time, a very strong direct evidence that proteolytic anti-MBP activity is intrinsic to IgGs from CSF of MS patients and compare some other parameters characterizing CSFs and sera of MS patients.

## Results

Fifteen patients (11 women and 4 men) satisfying the criteria for clinically or laboratory-supported definite MS according to [23], [24] were retrospectively selected for the study. Of these, 13 were relapsing–remitting (RR), and 2 were primary progressive (PP) in agreement with the criteria of Lublin and Reingold [25]. Clinical course (RR and PP), clinical activity (relapse at time of sampling), and MRI activity (the presence of gadolinium enhancing lesions at MRI examination) were analyzed as described previously [26]. The characteristics of the MS patients are summarized in Table 1.

**Table 1:** Several different characteristics of MS patients.

Number of patient	Sex	Age, yeas	Clinical course*	Clinical activity**	MRI activityξ
1	male	59	PP	yes	yes
2	female	28	RR	no	no
3	female	36	RR	yes	yes
4	male	26	RR	yes	no
5	male	49	RR	no	no
6	female	20	RR	yes	no
7	female	46	PP	yes	no
8	female	51	RR	yes	yes
9	female	31	RR	yes	no
10	female	26	RR	no	no
11	female	43	RR	yes	yes
12	male	45	RR	yes	no
13	female	30	RR	no	yes
14	female	60	RR	yes	no
15	female	34	RR	yes	yes

*Relapsing–remitting (RR) and primary progressive (PP) MS.

**Clinical activity  =  presence of relapse at the time of sampling.

ξ MRI activity  =  presence or absence gadolinium enhancing lesions at MRI examination.

It was interesting to compare some different indexes for CSF and sera of MS patients. Therefore, first we measured a relative concentration of total protein in CSF and sera of MS patients (Table 2). The relative concentrations of total protein of CSFs (range 0.26–0.66 mg/ml) and sera (47–74 mg/ml) of fifteen MS patients varied in different ranges. The average concentration of total protein in the serum (62.5±6.7 mg/ml) was ∼130-fold higher compared with CSF (0.48±0.09 mg/ml) and these values did not demonstrate a good correlation (coefficient correlation (CC)  = −0.12; p<0.05), Table 2). The relative concentration of total IgGs in the serum (range 7.87–16.6 mg/ml; average value (11.7±1.8 mg/ml) was 195-fold higher than that for CSF (range (1.9–13.6×10^−2^ mg/ml; average value (6.0±3.1)×10^−2^ mg/ml) and there was not a good correlation between these values, CC = 0.4 (p<0.05). Interestingly, the concentration of total protein in CSF was 8-fold higher than total IgGs, while this difference in the case of the serum (∼5.4-fold) was by a factor of approximately 1.5 lower.

**Table 2:** The relative content of different IgGs and anti-MBP antibodies in sera and CSF of patients with MS*.

Total protein (PR) or IgG type	Average values ±S.E. (mg/ml and % or A_450_)*					
	Serum		CSF		Ratio 1 and 3, (mg/ml)**	Ratio (%) of 2 and 4 (*p*), **
	Abs or PRΥ, mg/ml (1)	Abs, % (2)	Abs or PR, mg/ml (3)	Abs, % (4)		
Total protein	62.5±6.7	100	0.48±0.09	100	136	1.0
total-IgG	11.7±1.8	100	(6.0±3.1)×10^−2^	100	195	1.0
λ-IgG	1.4±0.07	12.3±0.6	(0.48±0.1)×10^−2^	8.0±1.7	292	1.54 (1.4×10^−6^)
κ-IgG	10.3±0.07	87.7±0.6	(5.52±0.1)×10^−2^	92±1.7	187	0.95 (1.2×10^−6^)
IgG1	6.3±1.2	53.6±10.6	(2.42±0.16)×10^−2^	40.4±2.7	260	1.3 (4.6×10^−4^)
IgG2	4.2±0.9	36.0±9.8	(2.94±0.18)×10^−2^	49.0±3.0	143	0.73 (3.7×10^−4^)
IgG3	0.66±0.14	5.6±1.2	(0.49±0.04)×10^−2^	8.2±0.6	135	0.68 (9.3×10^−6^)
IgG4	0.56±0.15	4.8±1.3	(0.15±0.02)×10^−2^	2.5±0.4	373	1.92 (1.5×10^−5^)
Anti-MBP Absξ	A_450_ = 0.32±0.08	100	A_450_ = (1.4±0.72) ×10^−3^	100	229	-

*The average values measured in the case of fifteen individual MS patients as mean ±S.E; for each value of individual patients a mean of three measurements was used; the error of the determination of these values did not exceed 7–10%.

**The ratio of the average values are reported; *p* is the Student *t* test criteria.

ξ Different dilution CSF (5-fold) and serum (100-fold) preparations were used; relative content was calculated for the same 100-fold dilution of CSFs and sera.

Υ PR, total protein in analyzed CSFs or sera.

**Figure 1:**
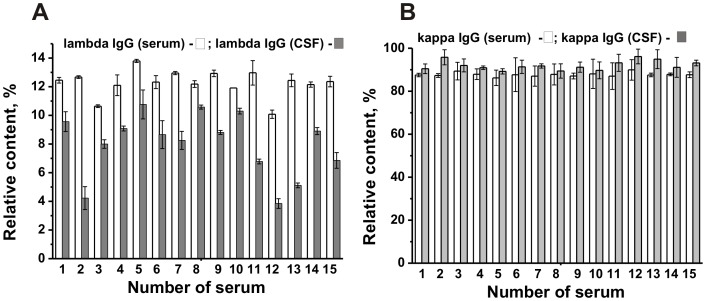
Relative content (%) of lambda- (A) and kappa (B) IgGs in the CSFs (▪) and sera (□) of fifteen MS patients. Total amount of IgGs in the CSFs and sera was taken for 100%. For details, see Materials and methods.

Then, we estimated the relative concentration of IgGs containing kappa and lambda light chains in serum and CSF. The relative content of λ-Abs in the sera of all patients was comparable (range 10.0–13.8% of total Abs; average value 12.3±0.6% or 1.4±0.07 mg/ml), while in the case of CSF it was remarkably varied (range 5.1–10.8%; average value 8.0±1.7% or (0.48±0.1)×10^−2^ mg/ml) (Fig. 1A), no good correlation was observed, CC = 0.40 (p<0.05). The relative content of κ-Abs in the sera was significantly higher (range 86.2–89.9%; average value 87.7±0.6% or 10.3±0.07 mg/ml), and a similar situation was observed in the case of CSF (range 89.7–96.2%; average value 92±1.7% or (5.5±0.1)×10^−2^ mg/ml). Overall, the relative content of λ-Abs (% of total Abs) in the sera was approximately 1.54-fold statistically significantly higher, while the content of κ-Abs was ∼1.1-fold statistically significantly lower than that for CSF (Table 2; *p* =  (1.2–1.4) ×10^−6^).

**Figure 2:**
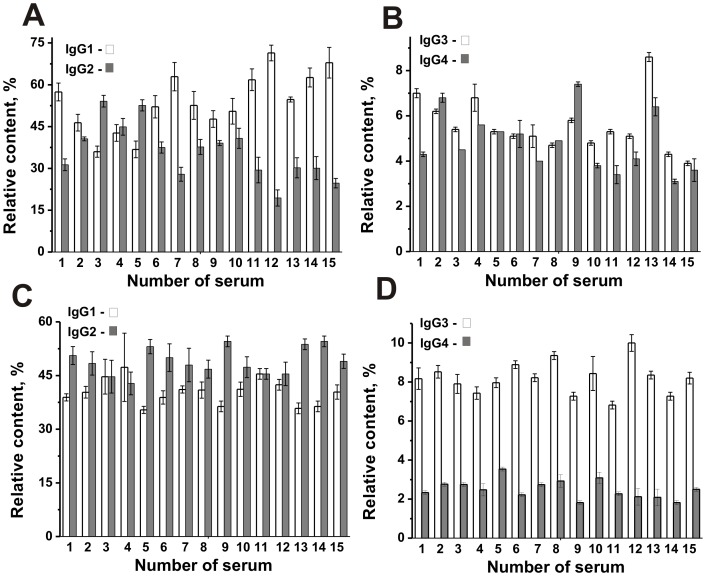
Relative content (%) of IgG1 and IgG2 (A), IgG3 and IgG4 (B) in sera, as well as IgG1 and IgG2 (C), and IgG3 and IgG4 (D) in CSFs of fifteen MS patients. Total amount of all types of IgGs in the CSFs and sera was taken for 100%. For details, see Materials and methods.

In the next step, we compared the relative content of IgG1, IgG2, IgG3, and IgG4 in CSF and sera (Fig. 2). The content of IgG1 (%) in fifteen CSF was to some extent comparable (range 35.4–47.3%; average value 40.4±2.7% or (2.42±0.16)×10^−2^ mg/ml) (Fig. 2B). The average relative content (%) of IgG1 in the sera was 1.3-fold statistically significantly higher than for CSFs (*p* = 4.6 ×10^−4^); these values for sera were significantly different for the various patients (range 36.0–71.4%; average value 53.6±10.6% or (6.3±1.2 mg/ml) (Fig. 2A, Table 2). At the same time, the average relative content of IgG2 was ∼1.4-fold statistically significantly higher (*p* = 3.7×10^−4^, Table 2) in CSF (range 42.8–54.5%; average value 49.0±3.0% or (2.94±0.18)×10^−2^ mg/ml; Fig. 2C) than in the sera (range 19.4–54.1%; average value 36.0±9.8% or 4.2±0.9 mg/ml; Fig. 2A) and the difference in values in sera was significantly higher in the various patients (Table 2).

The biggest difference in CSF and sera Abs was observed in the case of IgG3 and IgG4. The average content of IgG3 in the sera was 1.5-fold statistically significantly lower (*p* = 9.3×10^−6^), while IgG4 1.92-fold statistically significantly higher (*p* = 1.5×10^−5^) than those in the case of CSF (Fig. 2B and 2D). For all MS patients the content of IgG3 (range 6.8–10.0%; average value 8.2±0.6% or (0.49±0.04)×10^−2^ mg/ml) and IgG4 (range 1.8–3.5%; average value 2.5±0.4% or (0.15±0.02)×10^−2^ mg/ml) in CSF was different. In general, the content of IgG1, IgG2, IgG3, and IgG4 of different subclasses in the case of CSF (40.4, 49.0, 8.2, and 2.5%) and the sera (53.6, 36, 5.6, and 4.8%) decreased in different order. In the case of CSF, the content (%) of IgG2 and IgG3 was higher than that in the sera. The highest content of IgG1 was observed in sera, while IgG2 for CSF (Fig. 2). All data summarized in Table 2.

**Figure 3:**
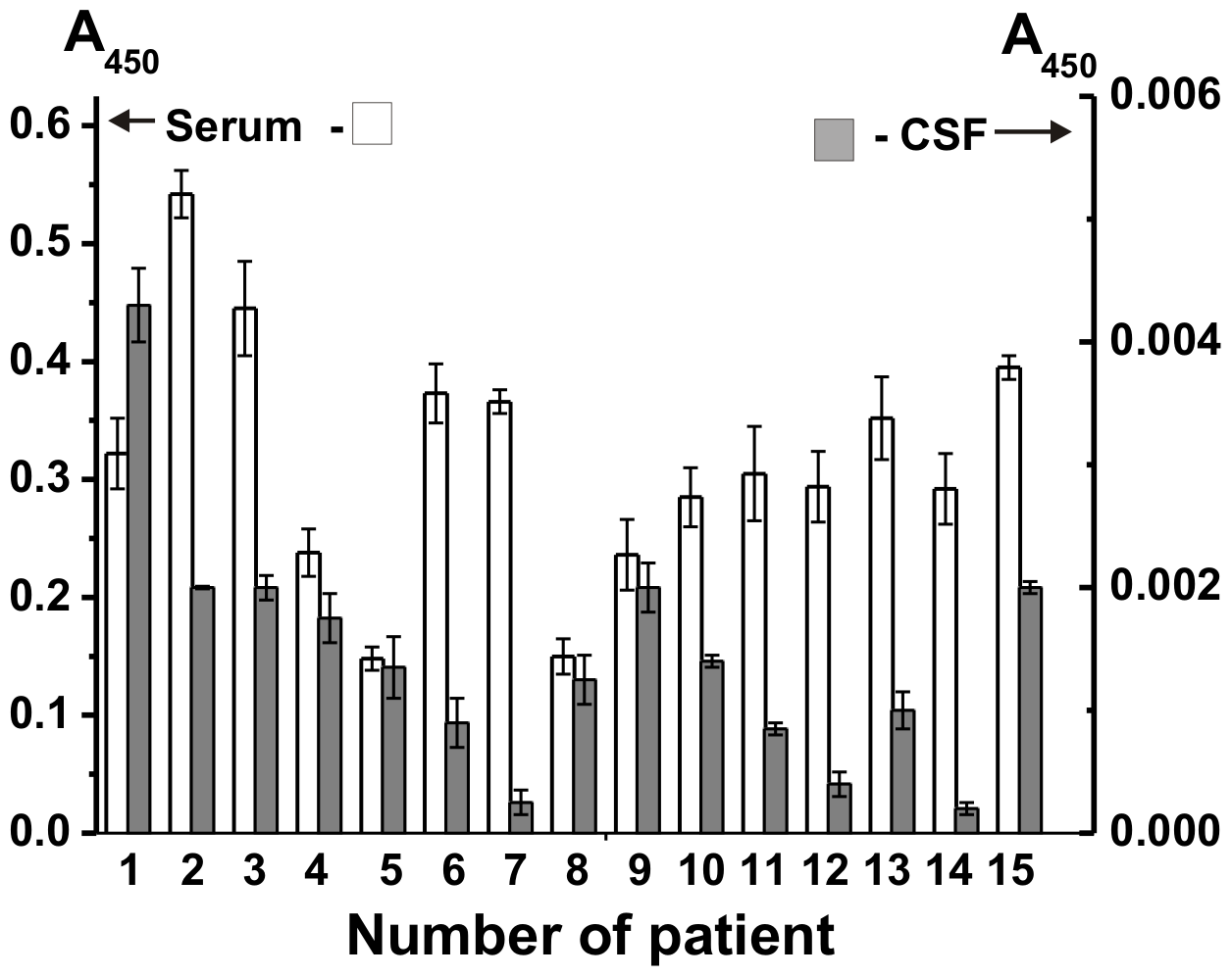
Relative content (A_450_) of anti-MBP IgGs in terms of the same 100-fold dilution for ELISA of CSF and serum preparations of fifteen MS patients. For details, see Materials and methods.

Serum anti-MBP Abs in sera of MS patients were reported in several articles [17], [27], [28], while there were no available data of these auto-Abs in CSFs of MS patients. We used ELISA to compare the relative levels of Abs against MBP in the sera and CSFs of 15 MS patients and seven healthy donors. Relative indexes of anti-MBP Abs in the sera of tested patients varied from 0.15 to 0.54, in average 0.32±0.08 A_450_ units (Fig. 3). In the case of seven healthy donors the relative indexes of anti-MBP Abs varied from 0.015 to 0.14 A_450_ and average value 0.08±0.05 A_450_ was approximately 4-fold lower than that for MS patients. These data was are in agreement with previously published results, according which the concentrations of auto-Abs against MBP in the sera of healthy donors is not zero and changed from 0.02 to 0.16 A_450_ units, in average 0.09±0.04 [17].

Using the same method and test system, it was shown that the indexes of anti-MBP Abs for CSF of the 15 MS patients in terms of the same dilution CSF and serum preparations, varied from 2×10^−4^ to 4.3×10^−3^ A_450_ units, in average (1.4±0.72)×10^−3^ A_450_ units (Fig. 3). We could not estimate real indexes of anti-MBP Abs in CSF of seven healthy donors, since A_450_ values in this case did not remarkably differ from background values. Thus, the average relative content of anti-MBP Abs in the sera of MS patients is approximately 230-fold higher than in the corresponding CSFs. At the same time the relative content of total IgGs in the sera is 195-fold higher than in the CSFs (Table 2). It means, that CSFs are on average approximately ∼1.2 depleted with Abs against MBP in relation to a total amount of antibodies compared to the same ratio of sera, and there is no good correlation between anti-MBP of CSFs and sera, CC = +0.02 (*p*<0.05).

**Figure 4:**
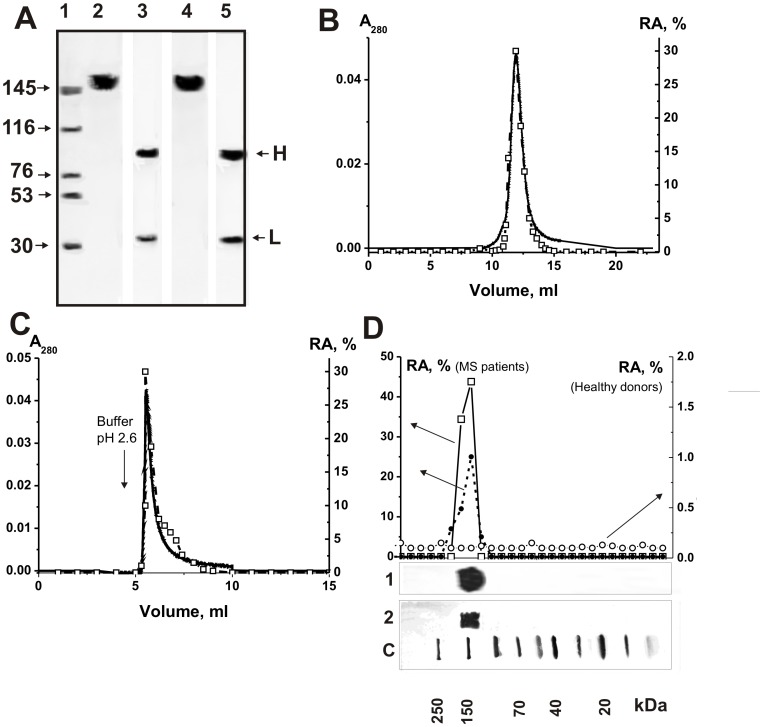
SDS-PAGE analysis of homogeneity of csf-IgG_mix_ (7 µg; lanes 2 and 3) and ser-IgG_mix_ (13 µg; lanes 4 and 5) corresponding to 15 CSFs and serum of MS patients in 3–16% gradient gel before (lanes 2 and 4) and after treatment with DTT (lanes 3 and 5) followed by silver staining (A). The arrows (lane 1) indicate the positions of molecular mass markers. FPLC gel filtration of csf-IgG_mix_ on a Superdex 200 column in an acidic buffer (pH 2.6) destroying immunocomplexes after Abs incubation in the same buffer (B) and csf-IgG_mix_ affinity chromatography on Sepharose bearing mouse IgGs against human IgGs (C): (—), absorbance at 280 nm (A_280_); (□), relative activity (RA) of IgGs in the hydrolysis of BMP. A complete hydrolysis of MBP was taken for 100%. In-gel assay of MBP-hydrolyzing activity of csf-IgG_mix_ (□; 15 µg) and ser-IgG_mix_ (•; 40 µg) of MS patients and csf-IgG_mix_ (○; 40 µg) (D). The relative MBP-hydrolyzing activity (RA, %) was revealed using the extracts of 2-3-mm fragments of one longitudinal slice of the gel. The RA of IgGs corresponding to complete hydrolysis of MBP was taken for 100%. The second control longitudinal slice of the same gel was stained with Coomassie Blue (lane 1, ser-IgG_mix_; lane 2, csf-IgG_mix_). Lane C shows positions of protein markers. The average error in the initial rate determination from three experiments did not exceed 7–10%. For details, see Materials and methods.

Recently we have demonstrated that highly purified IgGs from the sera of MS and SLE patients catalyze hydrolysis of MBP [17], [29], [30]. In this work, similarly to [17]–[20], [29], [30], electrophoretically and immunologically homogeneous IgGs were purified from CSFs and sera of MS patients and healthy donors by sequential chromatography on protein A-Sepharose under conditions that remove non-specifically bound proteins, followed by gel filtration in an acidic buffer destroying immune complexes. The homogeneity of the 150-kDa csf-IgG_mix_ and ser-IgG_mix_ (equal amounts of electrophoretically homogeneous IgGs from 15 preparations of CSFs and sera, denote these preparations as respectively csf-IgG_mix_ and ser-IgG_mix_) was confirmed by SDS-PAGE with silver staining, which showed a single band under non-reducing conditions and two bands corresponding to the heavy and light chains after reduction (Fig. 4A). Csf-IgG_mix_ and ser-IgG_mix_ corresponding to seven healthy donors were also electrophoretically homogeneous.

First, it was shown that all individual and mixed IgG preparations from CSFs (csf-IgG_mix_) and sera (ser-IgG_mix_) efficiently hydrolyze MBP (see below). Then, electrophoretically homogeneous csf-IgG_mix_ was used. To prove that MBP-hydrolyzing activity of csf-IgG_mix_ is an intrinsic property of MS Abs, we have applied several of known rigid criteria [11]–[14], [31]. The most important of these criteria are given below: a) electrophoretic homogeneity of csf-IgG_mix_ and ser-IgG_mix_ (Fig. 4A); b) FPLC gel-filtration of csf-IgG_mix_ under conditions of “acidic shock” (pH 2.6) did not lead to a disappearance of the activity, and the peak of proteolytic activity tracked exactly with 150 kDa IgGs (the proteolytic activity is absent in zones corresponding to molecular masses, 20–28 kDa, of known canonical proteases) (Fig. 4B); c) complete adsorption of the MBP-hydrolyzing activity by anti-IgG Sepharose leading to a disappearance of the catalytic activity from the solution and its elution from the adsorbent with buffer of acidic pH (Fig. 4C). Similar results were obtained for ser-IgG_mix_. It was shown previously, that IgG from sera of healthy donors cannot hydrolyze MBP [17]–[22]. Csf-IgG_mix_ and ser-IgG_mix_ corresponding to seven healthy donors analyzed in this article were also catalytically inactive after FPLC gel-filtration of Abs_mix_ under conditions of “acidic shock” and affinity chromatography on anti-IgG Sepharose.

To exclude possible artifacts due to hypothetical traces of contaminating canonical enzymes, the csf-IgG_mix_ and ser-IgG_mix_ preparations were separated by SDS-PAGE and its MBP-hydrolyzing activity was detected after the extraction of proteins from the separated gel slices (Fig. 4D). It is known that canonical proteases (trypsin, chymotrypsin, chymotrypsinogen etc) are enzymes of relatively low molecular masses, which are relatively easy to significant restore their enzymatic activity after their treatment with SDS. Since SDS dissociates any protein complexes, and the electrophoretic mobility of the usually low molecular mass proteases (∼20–30 kDa) cannot coincide with that of intact IgGs (or Ab complexes with proteases), the detection of proteolytic activity in the gel region corresponding only to intact IgGs from CSFs and sera, provides direct evidence that CSF and serum IgGs do not contain admixtures of canonical proteases and possess MBP-hydrolyzing activity. In addition, csf-IgG_mix_ and ser-IgG_mix_ corresponding to seven healthy donors were also inactive after SDS-PAGE electrophoresis (for, example Fig. 4D).

**Figure 5:**
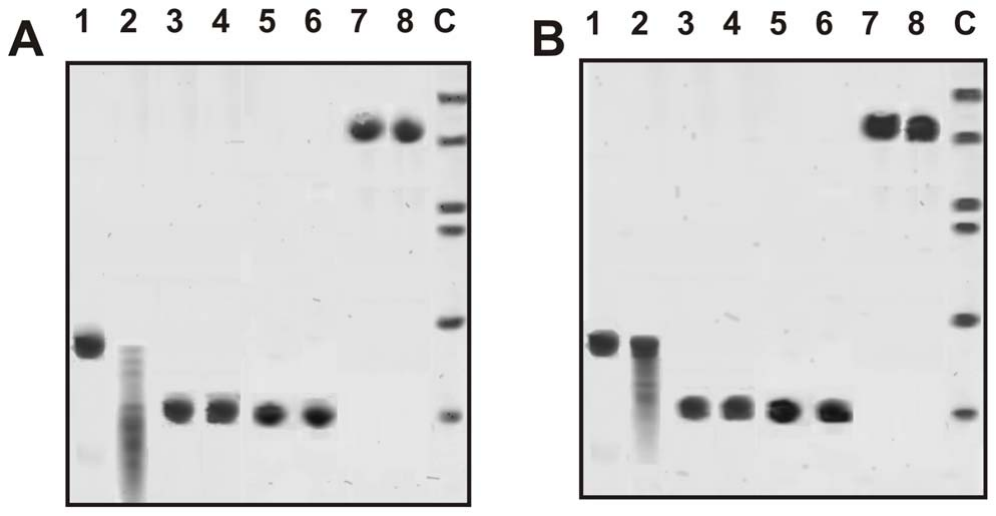
SDS-PAGE analysis of hydrolysis of various proteins by csf-IgG_mix_ (A) and ser-IgG_mix_ (B). Proteins (0.7 mg/ml) were incubated for 3 h without Abs (odd numbers), 0.03 mg/ml csf-IgG_mix_ (A) or 0.15 mg/ml ser-IgG_mix_ (even numbers): MBP (lanes 1 and 2), hen egg lysozyme (lanes 3 and 4); human milk lactalbumin (lanes 5 and 6), and human milk lactoferrin ((lanes 7 and 8) Lane C corresponds to a mixture of standard protein markers with known molecular masses.

It was shown previously, that polyclonal SLE and MS anti-MBP IgGs purified on MBP-Sepharose hydrolyze only MBP, but not many other tested proteins [17]–[20], [29]–[30]. Similar situation was observed in the case of abzymes against other proteins and peptides: HIV-1 reverse transcriptase and integrase, human serum albumin, casein, thyroglobulin, and intestinal vasoactive peptide [31]–[35]. However, IgG pools from the sera of patients with SLE and HIV-infected patients before their separation on sorbents bearing immobilized specific proteins can contain small fractions of abzymes hydrolyzing several various proteins including human serum albumin, casein, HIV reverse transcriptase and integrase [32]–[34]. Therefore, we could not exclude, that IgGs from CSFs and sera of some MS patients can contain small fractions of enzymes specifically hydrolyzing human serum albumin, casein, thyroglobulin, or some other proteins. At the same time, we did not find in the sera of various patients with autoimmune diseases abzymes hydrolyzing several other control proteins: hen egg lysozyme, human milk lactalbumin and lactoferrin [32]–[34]. Therefore, these three proteins, easily cleavable by canonical proteases, were used for analysis of csf-IgG_mix_ and ser-IgG_mix_ substrate specificity. Fig. 5A shows, that csf-IgG_mix_ efficiently cleave MBP, but cannot in the same conditions remarkably hydrolyze these three control proteins. Similar result was obtained for ser-IgG_mix_ (Fig. 5B). It is known, that classical mammalian, bacterial, and viral proteases are mostly unspecific and capable to hydrolyze any proteins or specific to bacterial and viral proteins. In contrast to canonical proteases, MS IgGs specifically hydrolyzed only MBP, but not other control tested proteins These data also indicate that IgG preparations from CSFs and sera do not contain impurities of classical human, bacterial, or viral proteases.

**Figure 6:**
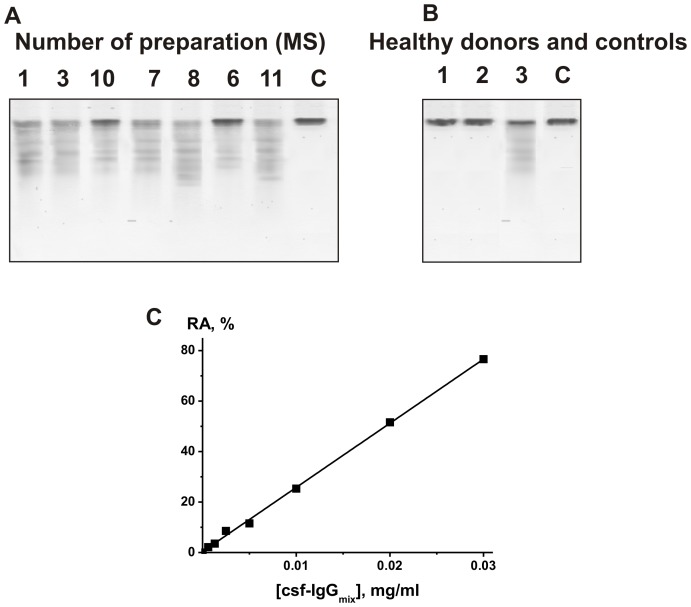
SDS-PAGE analysis of MBP-hydrolyzing activity of seven individual IgGs from different CSFs of MS patients (A) and healthy donors (B). The reaction mixtures were incubated with the IgGs 1 and 3 (0.02 mg/ml) or preparations 6, 7, 8, 10, and 11 (0.01 mg/ml) (A) as well as with the csf-IgG_mix_ (lane 1, 0.1 mg/ml) or ser-IgG_mix_ (lane 2, 0.1 mg/ml) of healthy donors (B); lane 2, csf-IgG_mix_ (0.01 mg/ml) of MS patients (B) for 3 h at 35°C. Lanes C (panels A and B) correspond to MBP incubated without Abs. Dependence of the relative MBP-hydrolyzing activity of csf-IgG_mix_ on its concentration (C). The average error in the initial rate determination from three experiments did not exceed 7–10%. For details, see Materials and methods.

It is known that due to alternative splicing of cDNA, animal and human MBP can consist of several related forms of different molecular masses (21.5, 18.5, 17.5, and 14.0 kDa) [36]. In addition, MBP may partially be hydrolyzed in human cells and during protein purification [37]. Therefore, highly purified preparations of MBP are usually not homogeneous and according to SDS-PAGE they can contain several protein bands, of which ∼18.5 kDa is usually major [37]. In this study we have used 18.5 kDa MBP containing no remarkable amounts of other forms of the protein. First, the relative activity of CSF Abs in the MBP cleavage was calculated from the decrease in the intensity of Coomassie-stained ∼18.5 kDa MBP band after electrophoresis as in [17]–[19]; the difference in the intensities of the protein, incubated in the absence and in the presence of Abs, was used for our calculations. To quantitatively estimate the protease activity, we have found a low concentration for each IgG preparation corresponding to the reaction of the first order, where the major ∼18.5 kDa MBP fraction is converted into products during hydrolysis within the linear regions of IgG concentration (for example, Fig. 6C demonstrates data for csf-IgG_mix_) and the time courses (15–40% of conversion; for example, Fig. 6A, lanes 3 and 6). IgGs from CSFs demonstrated high activity and we used low concentration of these Abs (0.005–0.03 mg/ml) and 2–5 h of incubation. Csf-IgG_mix_ and ser-IgG_mix_ (0.1 mg/ml corresponding to seven healthy donors were completely inactive in the hydrolysis of MBP (for example, Fig. 4D and 6B). Among 15 individual MS patients, the RAs of IgGs from CSFs at a fixed concentration of MBP (0.5 mg/ml) were very different (the specific RAs varied in a range 3.5–368 nmole MBP per hour per mg of Abs, while apparent *k_cat_* = V/[IgG] in the range 0.009–0.93 min^−1^); the average RA values were 175.0±94.5 nM MBP per hour per mg of Abs (average apparent *k_cat_* 0.4±0.26 min^−1^) (Table 3). The RAs of IgGs from sera were significantly lower and IgGs were used in higher concentrations (0.1–0.3 mg/ml), dependently of the preparation, the reaction mixtures were incubated for 8–20 h. The specific RAs of the sera IgGs varied in a range 0.21–7.5 nmole MBP per hour per mg of Abs and apparent *k_cat_* in the range (0.051–1.8 min^−1^)×10^−2^ min^−1^; the average values of RAs were 3.0±1.7 nM MBP per hour per mg of Abs (average apparent *k_cat_* (7.4±4.8)×10^−3^ min^−1^) (Table 3).

**Table 3:** The relative specific activities (RAs) and apparent catalytic constants characterizing IgGs from the CSFs and sera of different MS patients in the hydrolysis of MBP*.

Number of IgG	CSF		Serum		Ratio of RAs**
	RA, nmole MBP/1 h/mg	*k*_cat_, min^−1^ ‡	RA, nmole MBP/1 h/mg	*k*_cat_, min^−1^ ‡	
1	149.6	0.38	0.77	2.0×10^−3^	194
2	20.7	0.05	0.23	5.9×10^−4^	90
3	82.9	0.21	0.91	2.3×10^−3^	91
4	79.4	0.2	2.42	6.0×10^−3^	33
5	3.5	0.009	0.21	5.1×10^−4^	17
6	44.9	0.12	6.4	1.6×10^−2^	7
7	115.1	0.30	2.8	6.8×10^−3^	41
8	241.7	0.61	2.5	6.4×10^−3^	97
9	276.2	0.69	3.1	7.8×10^−3^	85
10	138.1	0.35	2.8	7.0×10^−3^	50
11	230.1	0.59	6.2	1.6×10^−2^	37
12	333.7	0.83	4.6	1.2×10^−2^	72
13	276.2	0.069	2.1	5.1×10^−4^	134
14	276.2	0.69	7.5	1.8×10^−2^	37
15	368.2	0.93	3.1	8.1×10^−3^	119
Average value	175.8±94.5	0.4±0.26	3.0±1.7	0.0074±0.0048	-

*The average values measured in the case of fifteen individual MS patients as mean ±S.E; for each value of individual patients a mean of three measurements was used; the error of the determination of these values did not exceed 7–10%.

**The ratios of the average values are reported.

‡ First, the relative specific RAs of every IgG preparation (nmole MBP/1 mg of protein/1 h) was calculated and then apparent *k*_cat_ values at fixed concentration of MBP (0.5 mg/ml) were calculated (*k*_cat_ = V/[IgG]).

It was surprising, but the average specific RA of IgGs from CSFs was about 58.6-fold higher than Abs from the sera. The ratio of specific RAs of MBP-hydrolyzing IgGs in the case of CSFs and serum of individual patients varied in the range 7 – 194-fold (Table 3). The coefficient of correlation (+0.42, *p*<0.05) between the anti-MBP hydrolyzing IgGs in the CSFs and sera is positive, but low. The CCs between the anti-MBP Abs titers (A_450_) and MBP-hydrolyzing activity in the case of CSF (−0.18, *p*<0.05) and sera (−0.1, *p*<0.05) were negative and also very low.

## Discussion

No analyses were made before any catalytic activities of Abs from CSF. Data reported in this paper provide strong evidence that MBP-hydrolyzing activity is an intrinsic property of IgGs present in CSF of MS patients: it is not due to copurifying enzymes. At entry none of the patients or donors had symptoms of infections. It was shown that CSFs and sera as well as IgGs after purification do not contain any bacterial contaminations.

Electrophoretically homogeneous csf-IgG_mix_ and ser-IgG_mix_ showed MBP-hydrolyzing activity after FPLC gel-filtration under conditions of “acidic shock”, affinity chromatography on anti-IgG Sepharose, and SDS-PAGE in zones corresponding only to intact IgGs; there was no activity corresponding to zones of canonical proteases having relatively low molecular masses (20–28 kDa; Fig. 4). In addition, in contrast to canonical proteases csf-IgG_mix_ and ser-IgG_mix_ did not hydrolyze control proteins: hen egg lysozyme, human milk lactalbumin and lactoferrin (Fig. 5).

Overall, abzymes of MS patients may be significantly more active in the hydrolysis of MBP than what we found (Table 3). As previously shown by us, the fraction of abzymes with different catalytic activities, including protease ones, in the serum of autoimmune patients usually does not exceed 1–7% of total immunoglobulins [11]–[14]. Since the specific activity was calculated using the total concentration of IgGs, the specific protease activities of the individual monoclonal subfractions in a polyclonal IgG pool may be significantly higher than those of the non-fractionated IgGs. In addition, the repertoire of polyclonal Abs against different antigens in the case of sera from MS patients may be significantly wider than that of CSFs. It may be one of the possible reasons of a lower specific activity of serum IgGs.

At the same time, an ever-growing number of observations suggest that autoimmune diseases originate from defects in hematopoietic stem cells [38]. It has recently been shown that the specific reorganization of the immune system during spontaneous development of a profound SLE-like pathology in MRL-lpr/lpr mice is associated with changes in the differentiation profile and the level of proliferation of bone marrow hematopoietic stem cells and with the production of DNase, ATPase, and amylase abzymes [39]–[41]. Immunization of healthy mice with DNA also leads to a production of Abs with DNase activity; however, it is only associated with increased lymphocyte proliferation and suppression of apoptosis of lymphocytes in different organs (especially in the spleen), but not with a change in the differentiation of bone marrow cells [39]–[41]. Thus, it is reasonable to suggest that B-cells of CSF of MS patients can produce not only Abs interacting with MBP, but also specific anti-MBP abzymes with high proteolytic activity. Abzymes produced by lymphocytes against MBP in different organs of MS patients (and circulating in the blood system) may have a lower MBP-hydrolyzing activity in comparison with anti-MBP Abs of CSF, or there may be different ratio of abzymes and anti-MBP Abs without catalytic activity in the CSFs and sera of MS patients.

We have not revealed high correlation coefficients between different indexes characterizing IgGs of CSF and sera of MS patients. Thus, an additional question is why there is no good correlation between various indexes, characterizing different MS patients. An analysis of correlation between titers of Abs to DNA as well as to MBP and 13 different standard clinical parameters including Poser criteria (indexes for evaluation of damage to functional systems: pyramidal functions; cerebellar functions; functions of brain stem; sensitive functions; functions of intestines and urinary bladder; visual functions; cerebral (psychical) functions and sum of these characteristics) in the case of 49 patients with MS was carried out [14]. For the whole group of MS patients, the absolute values of positive CCs between titers of anti-DNA or anti-MBP Abs and clinical Poser indexes were very low (between 0.01 and 0.19), absent (∼0), or even negative (−0.02 to −0.07) and statistically non-significant. Several CCs became higher and reached values up to 0.1 to 0.55 and −0.04 to −0.47 after the division of cohort into subgroups of patients with primary progressing, secondary progressing and remitting course of the disease [14].

The groups of primary progressing remitting course and secondary progressing course of MS patients were not “homogenous” with respect to the patients' characteristics, and their further subdivision using cluster and factorial analysis revealed high statistically significant correlation coefficients [14]. For example, for one sub-subgroup of the remitting course subgroup, a direct dependence between titers of anti-MBP and symptoms of lesions of the pyramidal tract could be observed (CC = 0.92). In some cases, correlations of the opposite sign were observed for the same pairs of analyzed parameters for the three subgroups with different MS courses and in their sub-subgroups obtained by cluster analysis from the subgroups.

The absence of a definite dependence between titers of anti-DNA and anti-MBP Abs and these parameters with standard clinical indices may be caused by several reasons. MS is an extremely multifactorial disease, in which similar pathomorphological and clinical indices manifested as MS may result from very different underlying processes and conditions [42], [43]. For example, in each MS patient, the “relative stability” of different organs and their functions to the destructive effect of transient immune system errors can be significantly different depending on the genetic background and environmental stress factors, including geographic ones [42]–[44]. Some proteins of influenza, herpes, polyoma, Epstein–Barr and other viruses and of some bacteria have been reported to mimic human myelin proteins, and these infections can therefore lead to immunization with their proteins and stimulate the subsequent formation of Abs to myelin and finally to the development of autoimmune reactions [45]–[48]. In individual MS patients, the development of autoimmune reactions can be stimulated by different viral or bacterial infections as well as various toxic chemicals. Furthermore, it should also be taken into account that MS is a pathology of at least two phases [48]. The cascade of reactions corresponding to the first inflammatory phase is very complicated and involves many proteins, enzymes, cytokines, and chemokines inducing macrophages and other cells producing NO^•^ radicals and osteopathin [43], [48]. The complex and coordinated action of T- and B-cells, complement system, inflammation mediators and auto-Abs result in the formation of demyelinization nodi and the interruption of axon conductivity. The neurodegenerative phase of MS that ensues thereafter is directly connected to the neural tissue destruction in these patients [43], [48]. Therefore, any analysis of biochemical, immunological and clinical indices must take into account the current stage of the disease. Obviously, quite different characteristics of pathologic processes can be obtained in individual patients as the disease progresses against the background of the continually changing immunoregulation, including the exhaustion of different compensatory and adaptive mechanisms and systemic metabolic changes. This makes the clinical course of MS hardly predictable in individual patients [43], [48]. Therefore, it is not surprising that we could not find a statistically significant correlation of titers of Abs to MBP and RAs of abzymes with all parameters measured, since each patient can be characterized by an individual combination of genetic, environmental, chronic, inflammatory, autoimmune, demyelinating, neurodegenerative and/or other factors.

In general, all data obtained demonstrate that the MBP-hydrolyzing activity is an intrinsic property of IgGs deriving from CSF and sera of MS patients. These IgGs are polyclonal and may consist of extremely different repertoires of protease subfractions in the case of CSF and sera. We have previously shown that the appearance of abzymes specifically hydrolyzing MBP is among the earliest and clear signs of autoimmune reactions in a number of autoimmune diseases when titers of Abs to MBP or other auto-antigens have not yet increased significantly and correspond to their ranges for healthy donors 11–14,39–41]. Therefore, detection of anti-MBP Abs with and without MBP-hydrolyzing activity in sera and CSF of people can be considered as an additional criterion (immunological parameter) for early diagnostics of MS.

## Methods

### Patients, donors and chemicals

Most chemicals, proteins, Protein G-Sepharose, and the Superdex 200 HR 10/30 column were from Sigma or GE Healthcare. We used purified human MBP containing 18.5 kDa form from RCMDT (Moscow; Russia). These preparations were free of lipids, oligosaccharides, nucleic acid, and other possible contaminations.

Fifteen consecutive MS patients (11 women and 4 men; mean age  = 39±12.5 years) satisfying the criteria for definite MS according to the classification of McDonald [23] and admitted to the Multiple Sclerosis Center of the University of Ferrara during the period from January 2012 to October 2012 were retrospectively selected for the study. Disease severity was scored in all MS patients at the time of sample collection using Kurtzke's Expanded Disability Status Scale (EDSS) [24] (mean at entry  = 1.8±1.4; range from 0 to 4.0). Clinical course (RR and PP), clinical activity (relapse at time of sampling), and MRI activity (the presence of gadolinium enhancing lesions at MRI examination) were analyzed as described previously [26]. In addition, we have used for control seven preparations of CSF and blood serum from healthy donors. At entry none of the patients or donors had fever or other symptoms or signs of acute infections. Moreover, at the time of sample collection none of the patients had received any potential disease-modifying therapies during the 6 months before the study.

### Sample preparation

The blood and CSF sampling protocols confirmed the local committee for medical ethics in research (Comitato Etico della Provincia di Ferrara) that approved our study in accordance with Helsinki ethics committee guidelines including written consent of patients confined to present of their blood and CSF for diagnostics of a possible disease and scientific purposes. The protocol was approved at 31 May 2007 and it was focused on the creation of a biological bank of CSF and serum samples, and related clinical data of patients with MS and other neurological diseases including: a) a study of potential markers (especially proteins) for diagnostic and prognostic significance in diseases of the nervous system; b) specific antibodies directed against antigens potential exogenous and/or endogenous; c) presence of pathogens (mostly viruses or bacteria) for association studies and pathogenesis; d) neurotransmitters and their metabolites; e) a study of different properties of different markers.

CSF and serum samples were collected under sterile conditions and stored in aliquots at –80°C until assay. “Cell-free” CSF samples were obtained after centrifugation, at room temperature, of specimens taken by atraumatic lumbar puncture performed for purposes of diagnosis in the absence of contraindications. Serum samples derived from the centrifugation of blood specimens withdrawn by puncture of an anterocubital vein at the same time of a CSF extraction. Paired CSF and serum samples from MS patients were stored and measured under exactly the same conditions. Informed consent was given by all patients before inclusion and the study design was approved by the Regional Committee for Medical Ethics in Research. CSF and serum IgG levels were measured by immunochemical nephelometry with the Beckman Immage 800 Immunochemistry System (Beckman Instruments, Inc. Fullerton, CA. USA) according to the procedure of Salden et al. [49].

### Analysis of protein concentrations

In all cases, protein concentration in the intact CSF, sera of MS patients and final solutions of Abs was measured using Bradford assay with a bovine serum albumin standard.

### Analysis of the concentration of total IgGs, lambda-, kappa IgGs, and IgGs of different subclasses

Relative concentrations of total IgGs in the intact CSF and in sera of MS patients were analyzed using a special quantitative isoelectrofocusing and immunoblotting test system according to the standard manufacturer's protocol and equipment (IgG IEF, Helena Laboratories, Gateshead, Tyne and Wear, UK). Relative concentrations of IgGs containing lambda and kappa type of light chains as well as IgGs of four different subclasses (IgG1-IgG4) were measured using special quantitative ELISA test systems from Vector Best (Russia). Horseradish peroxidase-conjugated with specific monoclonal mouse Abs against human IgGs (λ-IgGs, κ-IgGs, IgG1, IgG2, IgG3 and IgG4), and tetraethyl benzidine as substrate were used according to the standard manufacturer's protocol (Vector Best). Concentration of different IgGs (mg/ml) were estimated using calibration curves obtained according to the standard manufacturer's protocol.

### ELISA of anti-MBP autoantibodies

Anti-MBP auto-Abs were measured by ELISA. An optimization of all component concentrations, including buffers and time intervals of all operations for the achievement of a maximal difference between control and experimental samples, was carried out. Sodium carbonate buffer (50 µl, pH 9.6) containing 0.05 mg/ml MBP was added to ELISA strips and incubated overnight at 4°C. The assembled strips were washed four times with TBS buffer containing 0.01% NaN_3_ and 0.05% Triton X-100 and two times with the same buffer without Triton X-100. The block of strips surface was performed for 2 h at 37°C using TBS containing 0.2% bovine albumin, 0.05% NaN_3_. The strips were washed 10 times with water and then with TBS containing 0.01% NaN_3_. The preparations of human blood serum and CSF were diluted respectively 100 and 5 times with TBS containing 0.2% bovine albumin, 0.01% NaN_3_ and 0.05% Triton X-100 and 100 µl of final solution was added to the strips and incubated for 2 h at 37°C. After washing of the strips with water (10 times) and TBS, 100 µl TBS containing 0.2% bovine albumin and 0.01% NaN_3_ were added, incubation 2 h at 37°C. The strips were washed 10 times with water and incubated with 100 µl TBS containing 1 µg/ml conjugate of monoclonal anti-human IgG with horseradish peroxidase for 30 min at 37°C and washed again 10 times with water. After adding 50 µl citric-phosphate buffer containing 3,3′,5,5′-tetramethylbenzidine and H_2_O_2_ the strips were incubated for 15 min at room temperature and the reaction was stopped by adding of 50 µl of 50% H_2_SO_4_, and the optical density (A_450_) of the solutions was determined using a Uniskan II plate reader (MTX Lab Systems, USA). The relative concentrations of anti-MBP Abs the analyzed samples were expressed as the difference in the relative absorbance at 450 nm (average of three measurements) between the experimental samples and the control samples containing no Abs.

### IgG purification

Electrophoretically and immunologically homogeneous IgGs were obtained by sequential affinity chromatography of the CSF and serum proteins on protein A-Sepharose and FPLC gel filtration similarly to [17]–[20]. In order to protect the Ab preparations from bacterial contamination they were sterilized by filtration through a Millex filter (pore size 0.2 µm). In each case the protein corresponding to the central part of IgG peaks was concentrated in sterile condition and used in further purification or analysis. Incubation of standard bacterial medium with initial non-fractionated preparations of the sera, CSFs, and stored Ab preparations did not lead to a formation of colonies.

IgGs from CSF were incubated in 50 mM glycine-HCl (pH 2.6) containing 0.2 M NaCl for 20 min at 25°C. Separation of the IgGs under “acid shock” conditions was carried out by FPLC gel filtration on a Superdex 200 HR 10/30 column equilibrated with 50 mM glycine-HCl (pH 2.6) containing 0.1 M NaCl as previously described [17]–[20]. After 1–2 weeks of storage at 4°C, in order to refold Abs after the acid shock, these Abs were used in the activity assays described below.

In some cases, electrophoretically homogeneous IgGs were chromatographed on Sepharose bearing immobilized polyclonal mouse IgGs against human IgGs. The protein was applied to the column (1 ml) equilibrated with 20 mM Tris-HCl (pH 7.5) containing 0.1 M NaCl and the column was washed with the same buffer containing 0.3 M NaCl. Abs were eluted in 0.1 M glycine-HCl (pH 2.6), neutralized, dialyzed and sterilized as described above [17]–[20].

### Protein hydrolyzing activity assay

The reaction mixture (10–40 µl) for analysis of MBP-hydrolyzing activity of IgGs, containing 20 mM Tris-HCl (pH 7.5), 0.5–0.7 mg/ml MBP and 0.01–0.2 mg/ml of IgGs from CSFs or sera, was incubated for 2–20 h at 35°C. Hen egg lysozyme, human milk lactalbumin, and lactoferrin (0.7 mg/ml) were used for analysis of IgG substrate specificity; 0.03 mg/ml csf-IgG_mix_ or 0.15 mg/ml ser-IgG_mix_ were used. The MBP cleavage products were analyzed by SDS-PAGE in 12% or 4–15% gradient gels with Coomassie R250 staining. The gels were imaged by scanning and quantified using GelPro v3.1 software. The activities of IgG preparations were determined as a decrease in the percentage of initial MBP converted to different hydrolyzed forms in comparison with control MBP incubated without Abs. All measurements (initial rates) were taken under the conditions of the pseudo-first order of the reaction within the linear regions of the time courses (15–40% of MBP) and dependencies of MBP hydrolysis on IgG concentration.

### SDS-PAGE assay of proteolytic activity

SDS-PAGE analysis of Abs for homogeneity and for the polypeptide spectrum of the sera and CSF was performed in a 5–16% gradient gel containing 0.1% SDS (Laemmli system) as described in [17]–[20]. The polypeptides were visualized by silver and Coomassie Blue staining [17]–[20].

Analysis of MBP-hydrolyzing activity of MS IgGs from CSF and sera after SDS-PAGE was performed similarly to the analysis of the amylolytic and proteolytic activities of different abzymes [17]–[20], [50]. IgGs (10–40 µg) were pre-incubated at 30°C for 30 min under nonreducing (50 mM Tris-HCl, pH 7.5, 1% SDS, and 10% glycerol) condition. After standard SDS-PAGE electrophoresis of Abs to restore the MBP-hydrolyzing activity of IgGs, SDS was removed by incubation of the gel for 1 h at 30°C with 4 M urea and washed 10 times (7–10 min) with H_2_O. Then 2-3-mm cross sections of longitudinal slices of the gel were cut up and incubated with 50 µl 50 mM Tris-HCl, pH 7.5, containing 50 mM NaCl for 6 days at 4°C to allow protein refolding and eluting from the gel. The solutions were removed from the gels by centrifugation and used for assay of MBP hydrolysis as described below. Parallel control longitudinal lanes were used for detecting the position of IgG on the gel by Coomassie R250 staining.

### Statistical analysis

The results are reported as mean ±S.E. of at least three independent experiments for each sample analyzed. Errors in the values were within 5–10%. The correlation coefficients (CC) between sets of different samples were analyzed. The differences between samples were analyzed by the Student's *t*-test, *p*<0.05 was considered statistically significant.
